# Variants in *ARHGAP21*, encoding a Rho GTPase-activating protein, are associated with focal epilepsy and neurodevelopmental disorders

**DOI:** 10.1016/j.gendis.2025.101988

**Published:** 2025-12-15

**Authors:** Zilong Ye, Bingmei Li, Longshan Xie, Lingxia Fei, Xinguo Lu, Yunhua He, Jie Wang, Yongjun Chen, Weiping Liao, Yiwu Shi

**Affiliations:** a Department of Neurology, Institute of Neuroscience, Key Laboratory of Neurogenetics and Channelopathies of Guangdong Province and the Ministry of Education of China, The Second Affiliated Hospital, Guangzhou Medical University, Guangzhou, Guangdong 510260, China; b Foshan First People's Hospital, Foshan, Guangdong 528000, China; c Guangdong 999 Brain Hospital, Guangzhou, Guangdong 510510, China; d Shenzhen Children's Hospital, Shenzhen, Guangdong 518026, China; e The Affiliated Nanhua Hospital, Department of Neurology, Hengyang Medical School, University of South China, Hengyang, Hunan 421002, China

Rho GTPase-activating protein 21 (*ARHGAP21*) (MIM: 609870) encodes a member of the RhoGAP family that is predominantly expressed in the brain (https://www.proteinatlas.org/). The ARHGAP21 protein localizes to the Golgi apparatus via interaction with ADP-ribosylation factor 1 (ARF1) protein, where it inhibits cell division cycle 42 (CDC42) activity to regulate the actin-related protein complex 2/3 (ARP2/3) complex and actin dynamics. These processes are essential for maintaining Golgi structure and cytoskeletal organization, which are crucial for cell–cell junctions and adhesion.^1^ Previous studies have shown that variants in *ARHGAP21*, *ARF1*, and *CDC42* are associated with neurodevelopmental disorders (NDDs) and seizures/epilepsies (https://www.hgmd.cf.ac.uk/). While *ARF1* (MIM: 103180) and *CDC42* (MIM: 116952) are established causative genes, the pathogenic relevance of *ARHGAP21* variants in epilepsy/NDDs remains to be elucidated.

Here, we performed trio-based whole-exome sequencing and identified two *de novo* loss-of-function (LOF) and two compound heterozygous missense variants in *ARHGAP21* in four unrelated cases (five affected individuals) with focal epilepsy and/or NDDs (Fig. 1A). All variants were confirmed by Sanger sequencing (NM_020824.4; Fig. S1A), and no additional pathogenic/likely pathogenic variants were detected in known epilepsy/NDD genes. Variants p.Trp1337Ter and p.Glu1640Ter were absent in gnomAD, and the remaining four variants exhibited extremely low frequencies (MAF < 1 × 10^−5^; Table S1). All missense variants map to the disordered region of the ARHGAP21 protein (Fig. 1B), but display high conservation across diverse species (Fig. S1B), and are predicted to be damaging by more than five *in silico* tools (Table S1).

**Figure 1:**
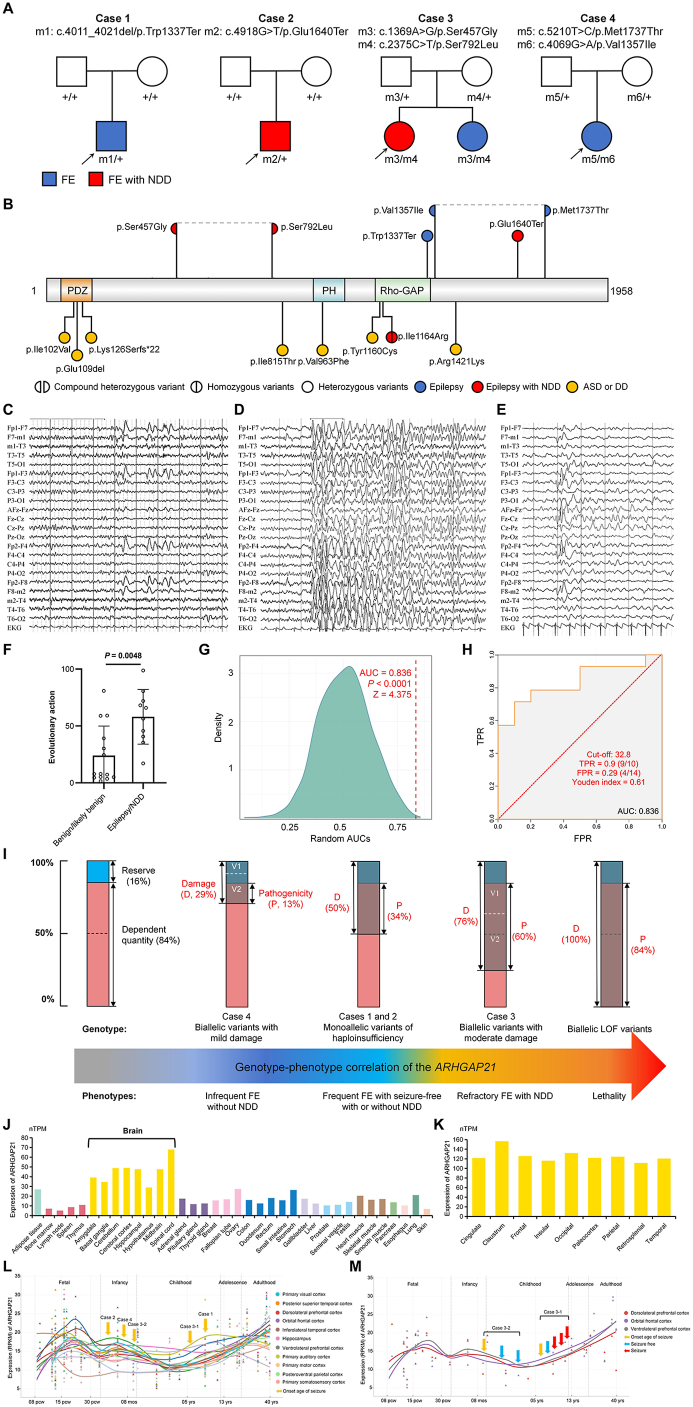
Variants in *ARHGAP21* are associated with focal epilepsy and neurodevelopmental disorders. **(A)** Pedigrees of the four unrelated cases with *ARHGAP21* variants. Individuals with variants are indicated by “m/+” or “m/m”, and those without variants are indicated by “+/+”. FE, focal epilepsy; NDD, neurodevelopmental disorder. **(B)** Schematic diagram of the ARHGAP21 protein and locations of the variants identified in this study (upper) and previous studies (lower). ASD, autism, DD, developmental disorder. **(C)** The interictal electroencephalography (EEG) of case 3-1 showed spike-slow waves in bilateral frontal regions at the age of 6. **(D)** The interictal EEG of case 3-1 showed diffuse slow activity at the age of 11. **(E)** The interictal EEG of case 3-2 showed spikes and spike-slow waves in the bilateral frontal regions at the age of 1. **(F)** The epilepsy/NDD-associated variants presented statistically higher evolutionary action (EA) scores than benign/likely benign variants. Data were calculated by the Mann–Whitney test. **(G)** Density plot of the random area under the curve (AUC). The red dashed line represents the observed AUC value. These metrics indicate that the observed AUC value is significantly higher than what would be expected randomly, suggesting the model has statistically significant effectiveness in distinguishing samples. **(H)** Receiver operating characteristic (ROC) curve. The cutoff value is 32.8, with a true positive rate (TPR = 0.9, 9/10), a false positive rate (FPR = 0.29, 4/14), and a Youden index of 0.61. These indicators demonstrate that the model performs well in distinguishing samples. **(I)** Genotype–phenotype correlation of *ARHGAP2*1 by understanding the relationship between the genetically dependent quantity (GDQ) and the damage of variants. The full scale of functioning of a gene in diploid individuals was set as 100% comprising a GDQ (the essential quantity required for normal function) and a Reserve. When the Damage of a variant is large enough to run out of the Reserve to produce a pathogenicity leading to an abnormal phenotype, and the severity of phenotypes is quantitatively correlated with the range of pathogenicity (*i.e.*, the Damage of variants beyond the Reserve). **(J, K)** RNA expression of *ARHGAP21* in human tissues (J) and different regions of the cerebral cortex (K), data obtained from the Human Protein Atlas datasets. nTPM, normalized transcripts per million. **(L, M)** The temporal expression pattern of *ARHGAP21* in multiple brain regions (L) and frontal regions (M). The expression levels were retrieved from the human RNA-sequencing data obtained from the BrainSpan database. The curve was fitted by the locally weighted scatterplot smoothing (LOWESS) method. Each arrow represents the patient's annual condition record, where the red arrow indicates seizure and the blue arrow indicates seizure-free. pcw, post-conceptual week; RPKM, reads per kilobase per million mapped reads; yrs, years.

The detailed clinical features of the patients are summarized in Table S2. Two boys (cases 1 and 2) with heterozygous LOF variants presented with frequent focal impaired awareness seizures (FIAS) or focal-to-bilateral tonic-clonic seizures (FBTC), occurring weekly/daily. Both responded well to antiseizure medications, with case 1 achieving seizure freedom on levetiracetam and case 2 on valproate. However, case 2 exhibited global developmental delay compared with their peers. Case 3-1 had occasional FBTC at the onset age of 6 years, then was seizure-free for approximately 1 year with levetiracetam, but recurred monthly FIAS at 8 years old and failed to control seizures with combined treatment of levetiracetam and lamotrigine. Her electroencephalogram showed evolution from focal frontal abnormalities (Fig. 1C) to diffuse multifocal ones (Fig. 1D). She had mild intellectual disability and significant memory decline. Her younger sister (case 3-2) with the same variants had only two FBTC, with focal frontal abnormal discharges (Fig. 1E), and has been seizure-free for 2 years on clonazepam with normal development. Case 4 experienced 2 FBTC, has been seizure-free for 4 years without antiseizure medications, and has no obvious intellectual/motor development disorders.

Based on gnomAD, *ARHGAP21* is shown to be highly LOF intolerant with a probability LOF intolerant (pLI) score of 1.0 and an observed/expected ratio of 0.19. *Arhgap21* homozygous knockout mice exhibited preweaning lethality, and heterozygous mice exhibited reduced lean body mass and abnormal auditory brainstem responses (https://www.informatics.jax.org/). Notably, biallelic LOF variants of *ARHGAP21*, including homozygous LOF and two heterozygous LOF variants *in trans*, were absent across the variant co-occurrence in the gnomAD database, suggesting that complete loss of *ARHGAP21* function may cause embryonic lethality in humans. Additionally, *ARHGAP21* is shown to have significant missense constraint with a Z-score of 4.19. These findings indicate that *ARHGAP21* would have a high genetically dependent quantity (GDQ, a required quantity of genetic function to maintain a gene biophysiological function; www.gdap.org.cn)^2^ or low damage tolerance (Reserve).

We next performed quantitative damage analysis of the variants to obtain the Reserve threshold of *ARHGAP21*. Variants associated with epilepsy/NDDs were classified as the disease group, including 8 previously reported likely pathogenic variants (Fig. 1B; Table S3), and the benign/likely benign variants from the gnomAD database as the control group (Table S4). Missense variants' damage scores were quantified using the evolutionary action (EA) equation,^3^ revealing significantly higher scores in the disease group compared with the control group (Fig. 1F). Subsequently, receiver operating characteristic curve analysis was applied to identify the optimal EA cutoff distinguishing the disease group from the control group. The area under the curve (AUC) was measured to be 0.836, significantly higher than expected by chance (*P* < 0.0001; Z = 4.375; Fig. 1G), indicating good discriminative ability. The optimal cutoff was determined to be 32.8 (Fig. 1H). Considering that each human gene has two alleles, the minimum GDQ of *ARHGAP21* in humans is approximately 84% (calculated as 1–32.8%/2), *i.e.*, if the damage of variants in *ARHGAP21* exceeds 16%, it would be pathogenic.

As shown in Figure 1I, the pathogenicity analysis (calculated as Damage–Reserve) was performed for all cases. The variant in case 3 showed the highest pathogenicity (60%, 76% Damage–16% Reserve), and the patient showed refractory focal epileptic seizures with NDDs. Case 4 showed the lowest pathogenicity (13%, 29% Damage–16% Reserve), and the patient showed the mildest symptoms and had seizure-free without antiseizure medications. Case 1 and case 2 had similar pathogenicity (34%, 50% Damage–16% Reserve), both exhibiting frequent seizures but good responsiveness to antiseizure medications. Together, these findings indicate a correlation between the pathogenicity of variants and phenotypic severity, with higher pathogenicity being associated with more severe clinical manifestations (Fig. 1I).

*ARHGAP21* has the highest expression level in the human brain (Fig. 1J) and is widely distributed across different regions of the cerebral cortex (Fig. 1K). Recent studies^4^ have indicated that the genetically dependent (expression) stage is correlated with the evolutionary process and age at disease onset. *ARHGAP21* is highly expressed in infancy and then decreases in early childhood but increases dramatically beginning in later childhood, which is consistent with the age of onset of the four patients (Fig. 1L). It is noted that case 3-1 and case 3-2 carried the same variant (p.Ser457Gly & p.Ser792Leu) but exhibited different clinical prognosis, *i.e.*, case 3-1 presented refractory epilepsy and case 3-2 presented only two focal seizures (Table S2). We then investigated the temporal expression of *ARHGAP21* in the frontal lobe (Fig. 1M), where EEG abnormalities originated in the two patients. Case 3-1 developed refractory epilepsy during stages of increased gene expression, whereas case 3-2 had achieved seizure-free status during stages of low gene expression; this temporal difference in the dynamics of gene expression may account for the distinct clinical outcomes of the two patients.

The protein–protein interaction network of the ARHGAP21 protein was further analyzed (STRING v12.0; https://cn.string-db.org/). Five high-confidence interacting partners of ARHGAP21 were identified (confidence score > 0.7; Fig. S2A), and all were confirmed to be associated with epilepsy/NDDs (Fig. S2B). Gene Ontology and Kyoto Encyclopedia of Genes and Genomes analyses revealed the potential roles of this gene in neurodevelopment-related biological processes and signaling regulation (Fig. S2C, S2D). Additionally, the Network Diffusion method^5^ was used to explore the functional relevance between *ARHGAP21* and known focal epilepsy genes from OMIM (https://www.omim.org/; Table S5). Random AUC distribution validation revealed a Z-score of 3.44 for the observed AUC (0.82; Fig. S2E, S2F), which was significantly greater than the random distribution, indicating a strong functional association between *ARHGAP21* and focal epilepsy.

In conclusion, this study suggests that *ARHGAP21* is a novel gene for focal epilepsy and/or NDDs. The gene shows a functional tolerance threshold of 16%, with variant pathogenicity correlating with clinical severity. Integrating temporal expression dynamics highlights the role of developmental regulation in phenotypic variability, offering new insights into precision medicine for genetic disorders.

## CRediT authorship contribution statement

**Zilong Ye:** Writing – review & editing, Writing – original draft, Project administration, Methodology, Investigation, Formal analysis, Data curation, Conceptualization. **Bingmei Li:** Resources, Investigation, Data curation. **Longshan Xie:** Resources, Formal analysis, Data curation. **Lingxia Fei:** Resources, Data curation. **Xinguo Lu:** Data curation. **Yunhua He:** Formal analysis. **Jie Wang:** Methodology. **Yongjun Chen:** Resources. **Weiping Liao:** Methodology. **Yiwu Shi:** Writing – review & editing, Writing – original draft, Supervision, Funding acquisition, Conceptualization.

## Data availability

The data that support the findings of this study are available from the corresponding author upon reasonable request.

## Ethics statement

This study followed the guidelines of the International Committee of Medical Journal Editors regarding patient consent for research or participation and received approval from the Ethics Committee of the Second Affiliated Hospital of Guangzhou Medical University (approval ethic number 2020-hs-49).

## Funding

This work was funded by the 10.13039/501100001809National Natural Science Foundation of China (No. 82171439 to Yiwu Shi), the Guangzhou Science and Technology Program City-University Joint Funding Project (China) (No. 2023A03J0412 to Yiwu Shi), and the “4310” program of clinical medical research of the 10.13039/501100009020University of South China (No. 20224310NHYCG11 to Yongjun Chen). The funders had no role in study design, data collection and analysis, and the decision to publish or prepare the manuscript.

## Conflict of interests

All the authors declared no conflict of interests.

## Figures and Tables

**Figure S1:**
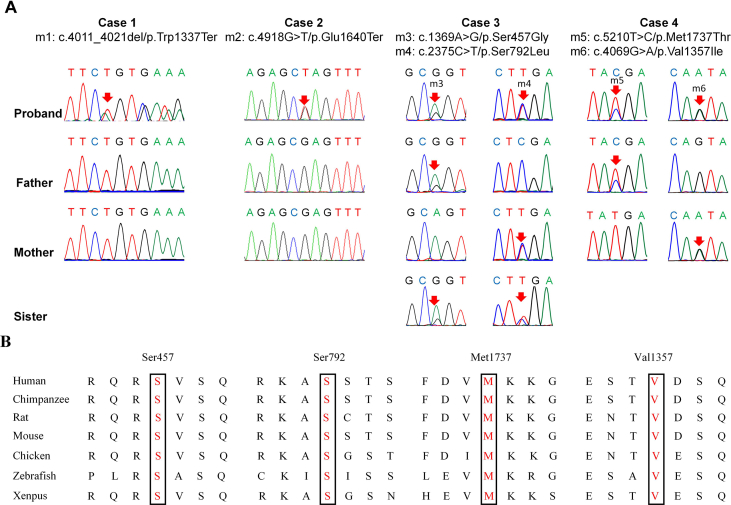
Genetic data and conservation analysis of patients with *ARHGAP21* variants. **(A)** DNA sequencing chromatogram of *ARHGAP21* variants. **(B)** Four residues, Ser457, Ser792, Met1737, and Val1357, are highly conserved across multiple species.

**Figure S2:**
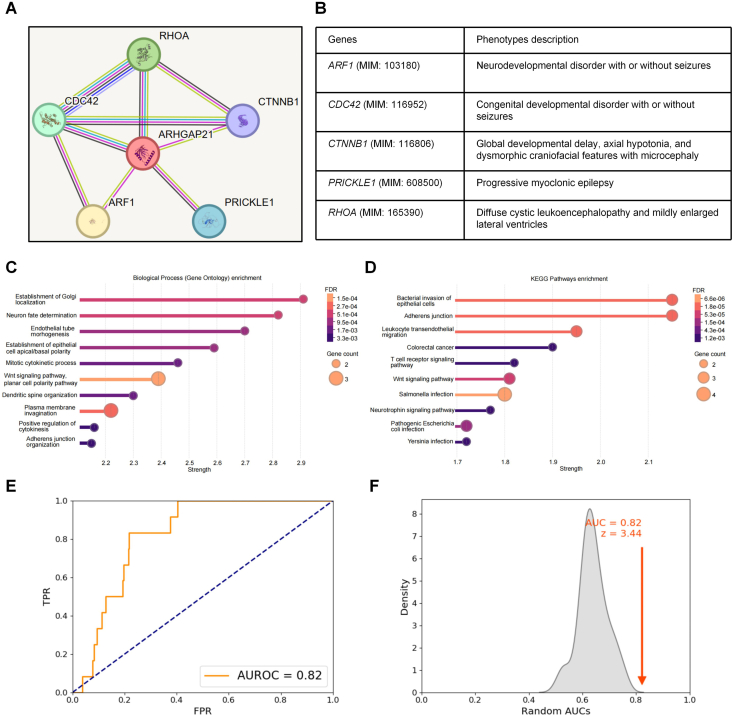
Protein–protein interaction network of *ARHGAP21* and functional links to focal epilepsy. **(A)** Protein–protein interaction network produced by STRING (version 12; confidence score >0.7). **(B)** The five genes that interact with *ARHGAP21* are all associated with neurodevelopmental disorders or epilepsy. **(C)** This figure illustrates the biological process enrichment analysis using Gene Ontology. Multiple biological processes (*e.g.*, establishment of the Golgi, neuron fate determination) are displayed, with the enrichment strength on the x-axis. The colour gradient represents the false discovery rate (FDR), and circle size corresponds to the gene count for each process. **(D)** This plot shows the KEGG pathway enrichment analysis. Various KEGG pathways (*e.g.*, adherens junction, Wnt signalling pathway) are presented, with enrichment strength along the x-axis. Color indicates FDR, and circle size reflects the gene count per pathway. **(E)** Receiver operating characteristic (ROC) curve. The area under the ROC curve (AUROC) was 0.82, indicating that the model had good performance in discriminating functional relevance. **(F)** Density plot of random AUCs. The actual AUC value (0.82) corresponds to a Z-score of 3.44, indicating the statistical significance of the observed AUC relative to random distributions and an association between *ARHGAP21* and focal epilepsy.
