# Supplementary material for: Variants in ARHGAP21, encoding a Rho GTPase-activating protein, are associated with focal epilepsy and neurodevelopmental disorders
Source: Genes Dis. 2025 Dec 15;13(5):101988. doi: 10.1016/j.gendis.2025.101988 (PMC13101686; doi:10.1016/j.gendis.2025.101988)
Supplement: Multimedia component 1 [file mmc1.doc]

**Supplementary Data**

**Supplementary Materials and Methods**

**Patients**

In this study, all cases (trios) with focal epilepsy without acquired etiologies were enrolled through the China Epilepsy Gene 1.0 Project, in which the cases were enrolled into 16 sub-cohorts based on clinical phenotype. Epilepsy syndromes were diagnosed and classified according to the criteria of the Commission on Classification and Terminology of the International League Against Epilepsy (2022). The inclusion criteria were as follows: (1) individuals without acquired causes, including brain tumors, head trauma, immune encephalitis, central nervous system infections, and cerebrovascular diseases, and (2) patients whose interictal electroencephalogram (EEG) typically showed focal epileptiform discharges.

This study adhered to the guidelines of the International Committee of Medical Journal Editors regarding patient consent for research participation and obtained approval from the Ethics Committee of the Second Affiliated Hospital of Guangzhou Medical University (ethics approval number: 2020-hs-49).

**Whole-exome sequencing and genetic analysis**

Blood samples were obtained from the probands and their parents. The detailed sequencing procedures used were described in previous studies^1-3^.

A case-by-case approach was used to identify candidate pathogenic variants in each case.^4-6^ Included variants in this study should meet the following criteria: (1) minor allele frequency (MAF) of < 0.005 according to gnomAD in the general population or East Asian population; (2) potentially pathogenic variants were retained, including canonical splice site, frameshift, initiation codon, in-frame, nonsense, missense, as well as intronic and synonymous variants predicted to affect splicing. The potentially pathogenic variants within each trio were analyzed using an individualized approach. Variants were filtrated according to the inheritance origin of each tri and variants with explainable genetic origins were retained. Then, a tiered MAF criterion was utilized to filter the variants:

(1) *De novo*, hemizygous, and homozygous variants were required to be absent in the gnomAD control population;

(2) For compound heterozygous variants, the product of the two alleles frequencies in gnomAD was set to < 1×10^-6^, a threshold sevenfold lower than the probability of observing such a genotype in the gnomAD population (1/141456 = 7 ×10^-6^).

Variants were finally filtrated, based on information on each gene summarized in Genetic Dependence & Pathogenicity Database (www.gdap.org.cn), by criteria on the gene profile of four aspects:

(1) Tissue-specific expression: the epilepsy candidate causative genes should be primally expressed in the brain (inclusion criteria). Concurrently, alternative pathogenic mechanisms—such as abnormal accumulation of metabolites or chronic toxicity—should be systematically evaluated to rule out their explanatory role in the disease phenotype.

(2) Exclude established causative genes with pre-existing gene-disease associations (i.e., genotype-phenotype correlations).

(3) The probability of being intolerant to heterozygous/homozygous variants of loss-of-function (pLI/pRec), genes of pLI ≥ 0.9 with *de novo* variants, and genes of pLI ≥ 0.9/pRec ≥ 0.9/pNull ≤ 0.1 with recessive variants were considered.

(4) Whether gene knockout/knockdown conditions produce relevant brain phenotypes.

*ARHGAP21* appeared as a candidate gene with recurrently *de novo* heterozygous and compound heterozygous variants. All *ARHGAP21* variants were confirmed by Sanger sequencing, and the reference transcript NM_020824.4 was annotated.

**Bioinformatic analysis**

Damaging effect: detailed predicted scores for *ARHGAP21* missense variants from nine commonly used *in silico* algorithms were obtained from the VarCards database (http://www.genemed.tech/varcards/) and used to preliminarily predict variant consequences.

Evolutionary Action (EA): EA equation is used to quantify the damaging effect of variants (<http://eaction.lichtargelab.org/>). Briefly, EA employs sequence homology and phylogenetic conservation to prioritize variants within each protein, scoring them on a 0–100 scale (0: benign, 100: pathogenic).^7-9^

Protein-protein interaction analysis: To analyse the interaction partners of the *ARHGAP21*, the STRING database (version 12.0, <https://cn.string-db.org/>) was used^10^. Setting the interaction score threshold for *ARHGAP21* at a minimum of 0.7 ensures high-confidence results.

Network Diffusion (nDiffusion): nDiffusion is a graph-based information diffusion analysis method that evaluates the functional relevance between two gene groups by simulating the spread of information from seed genes (with known functional annotations) to other nodes in biological networks. Operating under the assumption that closely connected genes in biological networks are more likely to share functions, nDiffusion integrates edge confidence weights (e.g., strength of experimental evidence) and multipath connectivity—rather than relying solely on the shortest path—to capture complex functional associations between genes more accurately. This approach outperforms traditional methods in prioritizing genes associated with the same pathways, functional categories, or diseases, combining speed and precision to enable functional interpretation of large-scale omics data and biological knowledge discovery.^11-13^ In this study, *ARHGAP21* and its five interacting genes were grouped together (Fig. S2A), while genes reported in OMIM as causative for focal epilepsy were used as the comparison group (Table S5). The online web-interface version of Network Diffusion was employed to explore potential functional associations between the two gene sets (http://ndiffusion.lichtargelab.org/), using default parameter settings, including step size, decay constant, and mode selection.

**Quantifying the damaging tolerant threshold of *ARHGAP21***

To quantify the damage tolerance threshold of *ARHGAP21*, we first systematically searched the PubMed and Human Gene Mutation Database (HGMD, http://www.hgmd.cf.ac.uk/ac/index.php). A total of 19 disease-related *ARHGAP21* variants were collected up to April 2025. Subsequently, we performed pathogenicity assessment using the criteria described in our genetic analysis above and identified 8 likely pathogenic variants associated with NDDs. Additionally, we collected all reported benign/possibly benign variants from gnomAD (v4.1.0) and obtained 15 variants. These variants were categorized into a disease group and a control group. The disease group included the 8 likely pathogenic variants associated with NDDs and 4 epilepsy-associated variants identified in this study, and the control group included the 15 benign/likely benign variants from gnomAD database. EA equation was used to quantify the damaging effect of the missense variants, including 10 missense variants in disease group and 14 missense variants in control group. Receiver operating characteristic (ROC) curve analysis was them performed to identify the optimal EA cutoff value between the disease group and the control group. Given that each human gene has two alleles, we processed the relevant data through percentage transformation and ultimately determined the damage tolerance threshold of *ARHGAP21* in humans.

**Expression analysis of *ARHGAP21***

RNA expression levels of *ARHGAP21* across multiple tissues were assessed using data from the Human Protein Atlas (HPA) dataset (https://www.proteinatlas.org/). Spatiotemporal expression patterns were investigated via the Brainspan database (http://www.brainspan.org/), which encompasses samples ranging from 8 post-conceptional weeks to 40 years. Expression spline was generated using the locally weighted scatterplot smoothing (LOWESS) algorithm to interpret the expression pattern of *ARHGAP21*.

**Statistical analysis**

Mann-Whitney test was used to assess nonparametric data. Receiver operating characteristic (ROC) curve analysis was conducted to identify the optimal EA cutoff value between the disease group and the control group. Statistical analyses were performed with R statistical software (V.4.2.2) and GraphPad Prism 8.3. *P* values less than 0.05 or Z score more than 2 were considered statistically significant.

**References**

1. Fan, C.X. *et al.* Heterozygous variants in USP25 cause genetic generalized epilepsy. *Brain* **147**, 3442-3457 (2024).

2. Ye, Z.L. *et al.* De novo heterozygous missense variants in ATP11A are associated with refractory focal epilepsy. *J Med Genet* (2025).

3. Ye, Z.L. *et al.* NEXMIF variants are associated with epilepsy with or without intellectual disability. *Seizure* **116**, 93-99 (2024).

4. Wang, J. *et al.* UNC13B variants associated with partial epilepsy with favourable outcome. *Brain* **144**, 3050-3060 (2021).

5. Ye, T. *et al.* Variants in BSN gene associated with epilepsy with favourable outcome. *J Med Genet* **60**, 776-783 (2023).

6. Shen, N. *et al.* Variants of TSC1 are associated with developmental and epileptic encep halopathy and focal epilepsy without tuberous sclerosis. *Acta Epileptologica* **6**.

7. Ally, A. *et al.* Comprehensive and Integrative Genomic Characterization of Hepatocellular Carcinoma. *Cell* **169**, 1327-1341.e23 (2017).

8. Shepherdson, J.L. *et al.* Variants in ZFX are associated with an X-linked neurodevelopmental disorder with recurrent facial gestalt. *The American Journal of Human Genetics* **111**, 487-508 (2024).

9. Katsonis, P. & Lichtarge, O. A formal perturbation equation between genotype and phenotype determines the Evolutionary Action of protein-coding variations on fitness. *Genome Research* **24**, 2050-2058 (2014).

10. Szklarczyk, D. *et al.* STRING v10: protein-protein interaction networks, integrated over the tree of life. *Nucleic Acids Res* **43**, D447-52 (2015).

11. Venner, E. *et al.* Accurate protein structure annotation through competitive diffusion of enzymatic functions over a network of local evolutionary similarities. *PLoS One* **5**, e14286 (2010).

12. Lisewski, A.M. *et al.* Supergenomic network compression and the discovery of EXP1 as a glutathione transferase inhibited by artesunate. *Cell* **158**, 916-928 (2014).

13. Pham, M. & Lichtarge, O. Graph-based information diffusion method for prioritizing functionally related genes in protein-protein interaction networks. *Pac Symp Biocomput* **25**, 439-450 (2020).
