# Supplementary material for: Variants in ARHGAP21, encoding a Rho GTPase-activating protein, are associated with focal epilepsy and neurodevelopmental disorders
Source: Genes Dis. 2025 Dec 15;13(5):101988. doi: 10.1016/j.gendis.2025.101988 (PMC13101686; doi:10.1016/j.gendis.2025.101988)
Supplement: Multimedia component 2 [file mmc2.docx]

**Supplementary Table 1. Genetic feature of the individuals with *ARHGAP21* variants**

| **Case no.** | **cDNA change** | **Protein change** | **Inheritance**  **pattern** | **MAF in controls** | **DANN** | **Fathmm_MKL** | **fitCons** | **GERP++** | **Polyphen-2_HDIV** | **Polyphen-2_HVAR** | **phyloP** | **SIFT** | **EA** | **ACMG** |
| --- | --- | --- | --- | --- | --- | --- | --- | --- | --- | --- | --- | --- | --- | --- |
| 1 | c.4011_4021del | p.Trp1337Ter | *De novo* | - | - | - | - | - | - | - | - | - | - | Path (PVS1+PS2+PM2+PP3) |
| 2 | c.4918G>T | p.Glu1640Ter | *De novo* | - | - | - | - | - | - | - | - | - | - | Path (PVS1+PS2+PM2+PP3) |
| 3 | c.1369A>G | p.Ser457Gly | Paternal | - | D (0.999) | D (0.995) | D (0.732) | C (5.86) | D (1) | D (0.997) | C (7.412) | T (0.376) | 71.97 | US (PM2+PP3) |
|  | c.2375C>T | p.Ser792Leu | Maternal | 1.25×10^-4^ | D (0.999) | D (0.986) | D (0.707) | C (5.25) | D (1) | D (0.999) | C (7.701) | D (0) | 80.65 | US (PM2+PP3) |
| 4 | c.5210T>C | p.Met1737Thr | Paternal | 1.83×10^-5^ | T (0.968) | D (0.978) | D (0.732) | C (3.55) | D (0.501) | B (0.081) | C (4.889) | D (0.001) | 17.06 | US (PM2+PP3) |
|  | c.4069G>A | p.Val1357Ile | Maternal | 1.83×10^-5^ | D (0.998) | D (0.989) | T (0.563) | C (4.6) | D (1) | D (0.996) | C (7.899) | D (0.002) | 40.70 | US (PM2+PP3) |

Abbreviations: ACMG, American College of Medical Genetics and Genomics; B, benign; C, conserved; DANN, combined annotation dependent depletion; D, damaging; EA, Evolutionary Action; GERP++, Genomic Evolutionary Rate Profiling; Fathmm_MKL, Functional Analysis through Hidden Markov Models–Multiple Kernels Learning; MAF, minor allele frequency from Genome Aggregation Database; phyloP, Phylogenetic *P*-values; Polyphen-2_HDIV, Polymorphism Phenotyping v2-HumanDiv; Polyphen-2_HVAR, Polymorphism Phenotyping v2-HumanVar; PVS1, null variant (nonsense, frameshift, canonical ±1 or 2 splice sites, initiation codon, or multi-exon deletion) in a gene; PM2, absent in population databases; PP3, multiple lines of computational evidence support a deleterious effect on the gene/gene product; PS2, *de novo* in a patient with the disease and no family history; SIFT, Sorting Intolerant from Tolerant; T, tolerable.

**Supplementary Table 2. Clinical features of patients with *ARHGAP21* variants in this study**

| **No.** | **Variants (NM_020824.4)** | **Sex** | **Age** | **Onset age** | **Seizure course** | **EEG** | **Brain MRI** | **Development** | **ASMs** | **Diagnosis** | **Prognosis** |
| --- | --- | --- | --- | --- | --- | --- | --- | --- | --- | --- | --- |
| 1 | c.4011_4021del/  p.Trp1337Ter | M | 11 yr | 9 yr | FIAS 1-2 times/wk for 3 mo | Spike-slow waves in right frontal and central regions | Normal | Normal | LEV | FE | Seizure free for 1 yr |
| 2 | c.4918G>T/  p.Glu1640Ter | M | 3 yr | 3 mo | FBTC 2-3 times/d for 6 mo | Spikes in left temporal | Normal | Mild DD | VPA | FE, NDD | Seizure free for 2 yr |
| 3-1 | c.1369A>G/  p.Ser457Gly c.2375C>T/  p.Ser792Leu | F | 12 yr | 6 yr | FBTC once at 6 yr; recurrent FIAS 2-3 times/mo at 8 yr for 4 yr | Spike-slow waves in bilateral frontal regions at 6 yr; normal at 7 yr; diffuse activity at 11 yr | Normal | Mild ID, memory decline | LEV, LTG | FE, NDD | Refractory |
| 3-2 | c.1369A>G/  p.Ser457Gly c.2375C>T/  p.Ser792Leu | F | 3 yr | 1 yr | FBTC 2 times | Spikes and spike-slow waves in bilateral frontal regions at 1 yr; normal at 2 yr | Normal | Normal | CNZ | FE | Seizure free for 2 yr |
| 4 | c.5210T>C/  p.Met1737Thr  c.4069G>A/  p.Val1357Ile | F | 5 yr | 6 mo | FBTC 2 times | Normal at age of 2 yr | Normal | Normal | Untreated | FE | Seizure free for 4 yr |

Abbreviations: ASMs, antiseizure medications; CNZ, clonazepam; d, day; DD, developmental disorder; EEG, electroencephalogram; F, female; FBTC, focal to bilateral tonic-clonic; FE, focal epilepsy; FIAS, focal impaired awareness seizure; LEV, levetiracetam; LTG, lamotrigine; ID, intellectual disability; M, male; mo, month; MRI, magnetic resonance imaging; VPA, valproate; wk, week; yr, year.

**Supplementary Table 3. Previous reported likely pathogenic variants of *ARHGAP21***

| **No.** | **cDNA**  **(NM_020824.4)** | **Protein** | **Inheritance** | **MAF in gnomAD controls** | **Reported phenotype** | **Evolutionary Action** | **Reference** |
| --- | --- | --- | --- | --- | --- | --- | --- |
| 1 | c.304A>G | p.Ile102Val | *De novo* | Absent | Autism spectrum disorder | 56.42 | (Fu et al.) |
| 2 | c.2444T>C | p.Ile815Thr | *De novo* | Absent | Autism spectrum disorder | 35.39 | (Iossifov et al.) |
| 3 | c.2887G>T | p.Val963Phe | *De novo* | Absent | Developmental disorder | 65.97 | (Turner et al.) |
| 4 | c.3479A>G | p. Tyr1160Cys | *De novo* | Absent | Developmental disorder | 68.2 | (Kaplanis et al.) |
| 5 | c.3491T>G | p.Ile1164Arg | Homozygous | Absent | Intellectual disability, cortical atrophy, colpocephaly, seizures | 98.73 | (Karaca et al.) |
| 6 | c.4262G>A | p.Arg1421Lys | *De novo* | Absent | Autism spectrum disorder | 45.73 | (Fu et al.) |
| 7 | c.326_328delAAG | p.Glu109del | *De novo* | Absent | Autism spectrum disorder | - | (Fu et al.) |
| 8 | c.377_378delAA | p.Lys126Serfs*22 | *De novo* | Absent | Autism spectrum disorder | - | (Fu et al.) |

Fu, J. M., et al. "Rare Coding Variation Provides Insight into the Genetic Architecture and Phenotypic Context of Autism." *Nat Genet* 54.9 (2022): 1320-31. Print.

Iossifov, I., et al. "The Contribution of De Novo Coding Mutations to Autism Spectrum Disorder." *Nature* 515.7526 (2014): 216-21. Print.

Kaplanis, J., et al. "Evidence for 28 Genetic Disorders Discovered by Combining Healthcare and Research Data." *Nature* 586.7831 (2020): 757-62. Print.

Karaca, E., et al. "Genes That Affect Brain Structure and Function Identified by Rare Variant Analyses of Mendelian Neurologic Disease." *Neuron* 88.3 (2015): 499-513. Print.

Turner, T. N., et al. "Sex-Based Analysis of De Novo Variants in Neurodevelopmental Disorders." *Am J Hum Genet* 105.6 (2019): 1274-85. Print.

**Supplementary Table 4. Benign and likely benign variants of *ARHGAP21* in gnomAD**

| **No.** | **cDNA**  **(NM_020824.4)** | **Protein** | **Evolutionary Action** | **No.** | **cDNA** | **Protein** | **Evolutionary Action** |
| --- | --- | --- | --- | --- | --- | --- | --- |
| 1 | c.1474A>G | p.Arg492Gly | 80.9 | 8 | c.5107C>T | p.Leu1703Phe | 59.15 |
| 2 | c.1529A>G | p.Asn510Ser | 5.24 | 9 | c.5254G>A | p.Gly1752Ser | 7.75 |
| 3 | c.2051C>G | p.Ser684Cys | 24.53 | 10 | c.5648T>C | p.Ile1883Thr | 1.53 |
| 4 | c.2156A>G | p.Gln719Arg | 7.3 | 11 | c.5785G>A | p.Val1929Met | 4.98 |
| 5 | c.2296G>A | p.Gly766Arg | 7.85 | 12 | c.1826G>A | p.Arg609Gln | 30.22 |
| 6 | c.3047G>C | p.Cys1016Ser | 58.94 | 13 | c.3361G>A | p.Gly1121Ser | 39.14 |
| 7 | c.4688T>C | p.Met1563Thr | 3.57 | 14 | c.5849G>C | p.Ser1950Thr | 4.60 |

**Supplementary Table 5. Known causative genes for focal epilepsy in OMIM**

| **Gene** | **Gene**  **MIM number** | **Phenotype** | **Phenotype**  **MIM number** | **Clinical synopsis** |
| --- | --- | --- | --- | --- |
| *CHRNA2* | 118502 | Epilepsy, nocturnal frontal lobe, type 4 | 610353 | Seizures, nocturnal, usually occur in clusters; EEG shows frontal lobe origin, etc. |
| *CHRNA4* | 118504 | Epilepsy, nocturnal frontal lobe, 1 | 600513 | Motor seizures, nocturnal, usually occur in clusters during dozing or on awakening; Ictal EEG showed partial seizures with frontal lobe origin, etc. |
| *CNTNAP2* | 604569 | Pitt-Hopkins like syndrome 1 | [610042](https://www.omim.org/entry/610042) | Seizures, early-onset; focal seizures; intellectual disability, moderate to severe, etc. |
| *DEPDC5* | 614191 | Epilepsy, familial focal, with variable foci 1 | 604364 | Seizure, focal or multifocal onset; Temporal lobe epilepsy; Frontal lobe epilepsy; Parietal lobe epilepsy, etc. |
| *GRIN2A* | 138253 | Epilepsy, focal, with speech disorder and with or without impaired intellectual development | 245570 | Rolandic epilepsy; EEG shows centrotemporal spike-wave discharges; Verbal dyspraxia; Learning difficulties, etc. |
| *LGI1* | 604619 | Epilepsy, familial temporal lobe, 1 | 600512 | Temporal lobe epilepsy; Simple partial seizures; Complex partial seizures, etc. |
| *KCNT1* | 608167 | Epilepsy nocturnal frontal lobe, 5 | 615005 | Seizures, focal, partial, motor; Seizures occur in clusters; Dystonic posturing, etc. |
| *NPRL2* | 607072 | Epilepsy, familial focal, with variable foci 2 | 617116 | Frontal lobe epilepsy; Nocturnal frontal lobe epilepsy; Temporal lobe epilepsy, etc. |
| *NPRL3* | 600928 | Epilepsy, familial focal, with variable foci 3 | 617118 | Frontal lobe epilepsy; Nocturnal frontal lobe epilepsy; Temporal lobe epilepsy, etc. |
| *SCN3A* | 182391 | Epilepsy, familial focal, with variable foci 4 | 617935 | Seizures, focal, well-controlled; Delayed psychomotor development, mild (in some patients), etc. |
| *TBC1D24* | 613577 | Epilepsy, rolandic, with paroxysmal exercise-induce dystonia and writer's cramp | 608105 | Seizures, focal, partial, often hemifacial; Rolandic sharp waves and spikes seen on EEG; Seizures, generalized, may occur, etc. |
| *TSC1* | 605284 | Tuberous sclerosis-1 | 191100 | Central nervous system manifestations include epilepsy, learning difficulties, behavioral problems, and autism. |
